# Dermatofibrosarcoma Protuberans Presenting as a Primary Breast Mass

**DOI:** 10.7759/cureus.46052

**Published:** 2023-09-27

**Authors:** Daniel Malek, Harris Alam, Lin Luo, Andrea Hong, Michele Edison

**Affiliations:** 1 Radiology, AdventHealth Orlando, Orlando, USA

**Keywords:** benign skin tumor, benign breast condition, breast ultrasonography, diagnostic mammography, dermatofibroma sarcoma

## Abstract

Dermatofibrosarcoma protuberans (DFSP) is an atypical soft tissue malignancy that affects the dermis and subcutaneous tissue. The cause of DFSP is not clearly understood. This report highlights a rare case of DFSP of the left breast. We report a case of an 18-year-old female with past medical history of type 1 diabetes mellitus, who presented to the breast imaging clinic with a six-month history of left breast lump and associated skin discoloration. The patient had a dedicated left breast ultrasound which showed an indistinct, oval, hyperechoic mass in the superficial breast, measuring 1.4 x 1.0 x 2.5 cm with mild internal vascularity. An ultrasound-guided biopsy of this left breast mass was recommended and performed approximately three weeks later, demonstrating DFSP. The patient was then advised for consultation with Oncology, Surgical Oncology, and Radiation Oncology, to which surgical excision was the final recommendation. The patient had a wide local surgical excision procedure for her left breast mass with surgical pathology confirming negative margins shortly thereafter. This case highlights a great index of suspicion that should be taken when evaluating palpable breast masses with associated skin discoloration in young patients.

## Introduction

Dermatofibrosarcoma protuberans (DFSP) is an atypical soft tissue malignancy that affects the dermis, subcutaneous fat, fascia, and muscle [1]. It typically presents as a slowly growing, erythematous, slightly raised plaque, mainly found on the trunk, extremities, head, or neck. DFSP accounts for approximately 1% of all soft tissue sarcomas and less than 0.1% of all malignancies. It has a total annual incidence of 0.8-4.5 cases per million [1]. It typically occurs during the third and fifth decades of life and has a high prevalence among those of African American origin [2]. About 10% of cases report trauma to the area prior to tumor growth [3]. Multiple studies indicate that most DFSPs have a t(17:22) chromosomal translocation, which leads to upregulation of platelet-derived growth factor and eventual tumor formation [1]. The prognosis is good with a 10-year survival rate of 99.1%, as the tumor rarely metastasizes. Definitive diagnosis is made by either punch or excisional biopsy. On computed tomography (CT) and magnetic resonance imaging (MRI) scans, DFSP can show as a nodular, noncalcified mass [4]. The initial diagnostic imaging evaluation includes a radiograph of the chest, mammogram, and axillary ultrasound to evaluate for lymphadenopathy [5]. Contrast-enhanced magnetic resonance imaging (MRI) of the breast is used after histologic confirmation of DFSP to determine the extent of disease [5]. Treatment is with surgical excisions with 2 cm margins; recurrence rates are high due to incomplete removal [6]. After surgical excision, frequent follow-up imaging should be considered due to the risk of local recurrence. In this report, we present an 18-year-old patient who incidentally found an abnormal growth and discoloration on her left breast. She monitored it for over a year before seeking medical attention. 

## Case presentation

We report a case of an 18-year-old female with a past medical history of type 1 diabetes mellitus. The patient has no surgical history, is a nonsmoker, nondrinker, and has no family history of malignancy. The patient noticed a chronic discoloration located in the upper outer quadrant of her left breast for over a year. The skin discoloration eventually formed a palpable lump, and after six months, she sought medical attention. At the gynecologist clinic, the breast physical exam was pertinent for a 2 cm mass noted at the 2 o'clock position of the left breast, there was an overlying area of purplish discoloration of the skin. No right breast physical finding. All other physical exam findings were within normal limits which included cardiovascular, musculoskeletal, respiratory, abdominal, and neurological examination. She was later referred to the breast imaging clinic by her gynecologist. 

**Figure 1:**
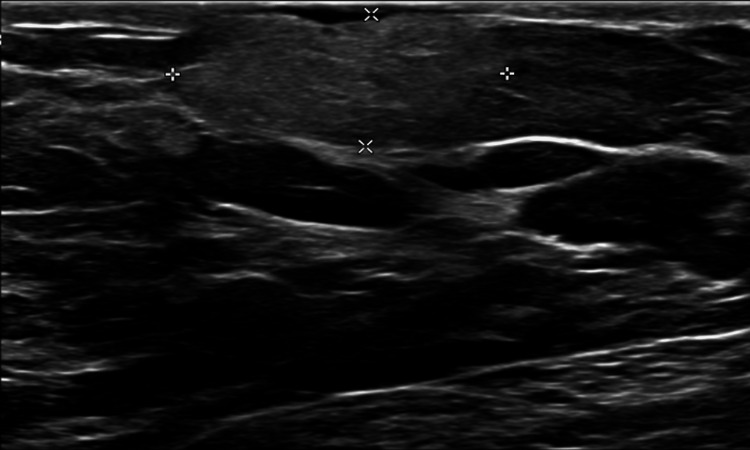
Gray-scale ultrasound image of the left breast upper outer quadrant palpable lump at the 2:00 axis, 8 cm from the nipple, demonstrates an indistinct, oval, parallel, hyperechoic mass. cm - centimeters Four (4) white measurements which highlight the axial and transverse measurements of the lesion.

**Figure 2:**
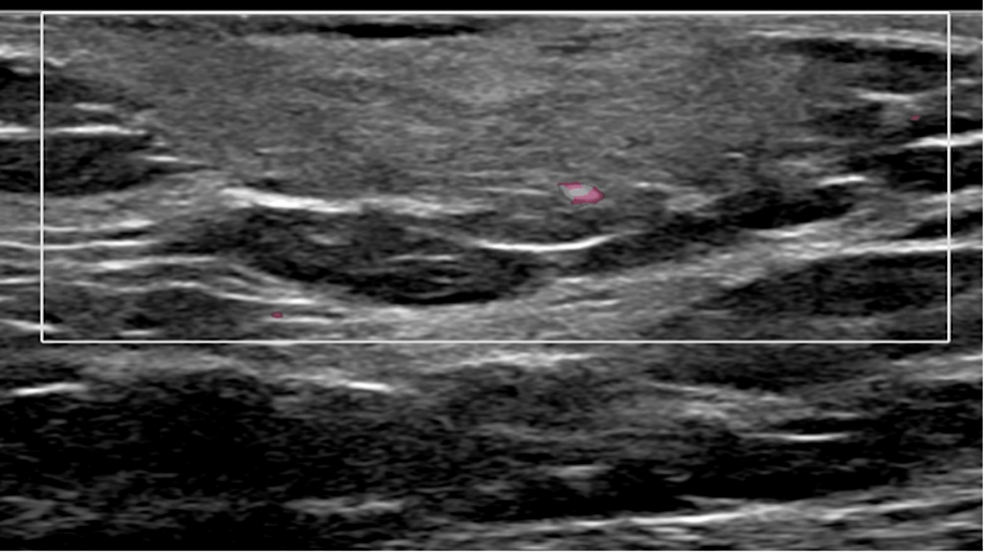
Color Doppler ultrasound image of the left breast palpable mass demonstrates mild internal vascularity.

The patient had a left breast ultrasound which showed an indistinct, oval, hyperechoic mass just deep to the skin, measuring 1.4 x 1.0 x 2.5 cm with mild internal vascularity (Figures 1, 2). The patient was recommended to undergo an ultrasound-guided core needle biopsy of this left breast mass. The pathology report of the biopsy returned positive for DFSP, sections showed spindle cell neoplasms with storiform patterns with borders involving fibro-adipose tissue. Immunohistochemical stains also show CD34-positive cells, supporting the diagnosis of dermatofibrosarcoma protuberans. The patient declined any genetic testing. Management options were discussed at our multidisciplinary breast tumor board, and it was decided that the patient should have wide local surgical excision with possible adjuvant radiation therapy if there were positive surgical margins.

**Figure 3:**
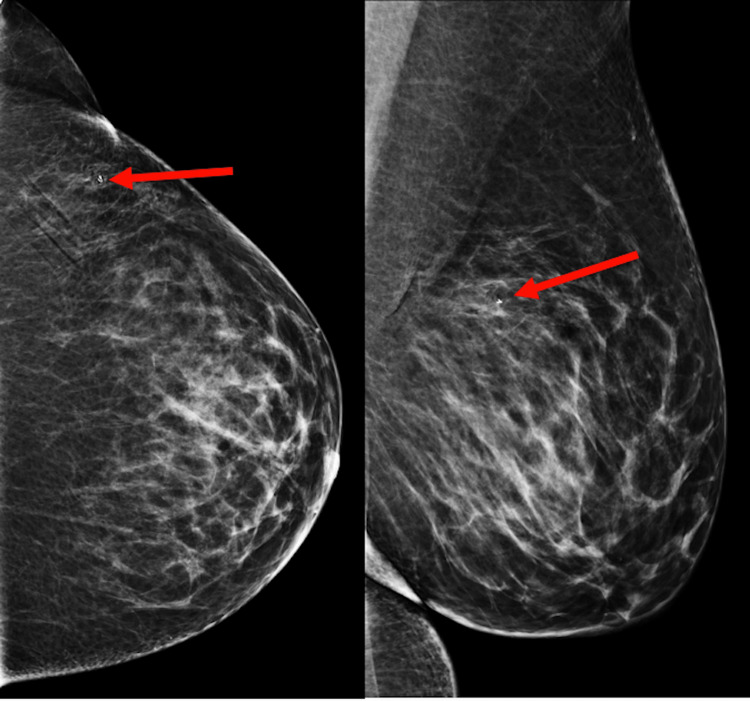
Left image CC and right image ML mammographic views demonstrates a heart-shaped titanium biopsy clip within a focal asymmetry in the upper outer quadrant of the left breast, confirming appropriate sampling. Red Arrows - Indicate heart-shaped biopsy clip within the focal asymmetry. CC - Craniocaudal view ML - Mediolateral view

Prior to surgery, breast examination showed a 28 mm firm mass in the upper outer quadrant of the patient’s left breast. There was no peau d’ orange, edema, or induration. Wide excision of the left breast with an intraoperative frozen section was performed, with confirmation of the desired 2 cm margins (Figure 3). At postoperative evaluation, the patient denied erythema, pain, tenderness, or drainage from the area. She continues to follow up with her oncologist to monitor for local recurrence for a total of five years as that is within the time frame for DFSP to re-occur.

## Discussion

This case highlights the great degree of caution that should be exercised when evaluating unusual breast masses. There have only been a few cases of dermatofibrosarcoma of the breast that have been reported in the literature [7]. DFSP has a high local recurrence rate due to the strong ability to invade local subcutaneous tissue. Most recurrences are detected within three years of primary excision. The five-year and 10-year survival rate of patients with local DFSP is above 99%. Treatment of localized DFSP is wide local excision with surgical margins of 2-3 cm and three-dimensional resection including skin, subcutaneous tissue, and underlying fascia [8]. Mohs surgery has been suggested to be the first-line therapeutic measure for optimal cosmesis (of the breast) and to reduce the recurrence rate [8]. Clinical and imaging follow-up is recommended for at least five years for early detection and management of potential local recurrence [9]. 

Sonographically, DFSP usually presents as a hypoechoic, circumscribed, oval mass [9]. Sometimes the mass may have peripheral vascularity on color Doppler ultrasound, as seen in Figure 2. Ultrasonography evaluation for DFSP can be non-specific. One study that evaluated four different patients demonstrated that in two cases of DFSP, the masses were hypoechoic and two had mixed echogenicity [10]. In addition, the color Doppler sonography had different blood flow patterns, one in which profuse blood flowed through the entire tumor and another where only a small amount of blood flowed through the peripheral portions of the tumor. DFSP on sonographic imaging is nonspecific, and can be easily confused with other superficial masses such as lipoma, epidermal cysts, and dermatofibroma [11].

Although this case report and most literature report dermatofibrosarcoma having prevalence in the breast and axilla there are other locations in which this may occur. Areas include the head and neck with a prevalence of 10-16% and the extremities with a prevalence of 20-30% [12]. Metastasis due to dermatofibrosarcoma is very rare, approximately 1-4% of cases. The majority of metastatic cases have been associated with local disease recurrence and poor prognosis [13]. Patients who are affected with metastatic dermatofibrosarcoma die within two years of diagnosis. It is recommended for patients to have strict long-term follow-up every six to 12 months with ultrasound evaluation for suspected recurrence, and biopsy if warranted [13]. Clinical diagnosis is often difficult, the tumor may show vague discoloration often brown or red, and maybe an individual nodule or clustered [14]. Dermatofibrosarcoma is a rare malignancy, and its occurrence on the breast is even more rare [15]. 

## Conclusions

In conclusion, DFSP of the breast is rare and may resemble other benign breast conditions, such as lipoma or fat necrosis. A high level of suspicion should be taken by clinicians when approaching atypical breast findings. Our patient’s age and clinical history prompted further evaluation. Through diagnostic ultrasound and subsequent ultrasound-guided biopsy, the patient was treated in a timely manner without significant associated morbidity.
